# Visual Perceptual Load Does Not Affect the Frequency Mismatch Negativity

**DOI:** 10.3389/fpsyg.2019.01970

**Published:** 2019-08-27

**Authors:** Stefan Wiens, Erik van Berlekom, Malina Szychowska, Rasmus Eklund

**Affiliations:** Gösta Ekmans Laboratory, Department of Psychology, Stockholm University, Stockholm, Sweden

**Keywords:** mismatch negativity, perceptual load, crossmodal attention, oddball, working memory capacity, N1

## Abstract

The mismatch negativity (MMN) has been of particular interest in auditory perception because of its sensitivity to auditory change. It is typically measured in an oddball task and is computed as the difference of deviant minus standard tones. Previous studies suggest that the oddball MMN can be reduced by crossmodal attention to a concurrent, difficult visual task. However, more recent studies did not replicate this effect. Because previous findings seem to be biased, we preregistered the present study and used Bayesian hypothesis testing to measure the strength of evidence for or against an effect of visual task difficulty. We manipulated visual perceptual load (high and low load). In the task, the visual stimuli were identical for both loads to avoid confounding effects from physical differences of the visual stimuli. We also measured the corrected MMN because the oddball MMN may be confounded by physical differences between deviant and standard tones. The corrected MMN is obtained with a separate control condition in which the same tone as the deviant (critical tone) is equiprobable with other tones. The corrected MMN is computed as deviant minus critical tones. Furthermore, we assessed working memory capacity to examine its moderating role. In our large sample (*N* = 49), the evidential strength in support of no effect of visual load was moderate for the oddball MMN (9.09 > BF01 > 3.57) and anecdotal to moderate for the corrected MMN (4.55 > BF01 > 2.17). Also, working memory capacity did not correlate with the visual load effect on the oddball MMN and the corrected MMN. The present findings support the robustness of the auditory frequency MMN to manipulations of crossmodal, visual attention and suggest that this relationship is not moderated by working memory capacity.

## Introduction

In a constantly changing world, humans need to monitor the auditory environment for events that may be dangerous or goal relevant, even if attention is engaged in other tasks. The mismatch negativity (MMN) has been of particular interest in auditory perception because of its sensitivity to auditory change (Näätänen et al., 1978; Kujala et al., 2007; Winkler, 2007; Näätänen and Kreegipuu, 2011; Fishman, 2014). It is typically measured in an *oddball* task (Duncan et al., 2009), in which a sequence of identical sounds (*standards*) is occasionally interrupted by different sounds (*deviants*). In electroencephalography (EEG), the oddball MMN is an event-related potential (ERP) that is obtained by taking the mean wave to deviants minus the mean wave to standards (i.e., deviant – standard). It has a frontocentral negativity with a latency of 150 to 200 ms after tone onset. The term MMN refers to the scalp-recorded negativity, and it is thought to index detection of change in a regular sequence of sounds (Fishman, 2014; Sussman et al., 2014).

In clinical research, the oddball MMN is widely used because it provides an unobtrusive index of auditory discrimination ability: If subjects show an MMN, then their auditory system is able to discriminate between deviant and standard (Duncan et al., 2009). However, deviant tones may also elicit the N2b that is obtained when attention is captured by the tones (Näätänen and Kreegipuu, 2011). Because this ERP response has a similar latency and topography as that of the MMN, these two responses may be difficult to separate. To minimize the confounding effects of N2b, subjects are typically instructed to watch a silent movie and ignore the tones (Duncan et al., 2009). In support, numerous previous studies have found that the MMN can be obtained when subjects read a book or watch a silent movie (Alho et al., 1992, 1994; Dittmann-Balcar et al., 1999; Kathmann et al., 1999; Harmony et al., 2000; Otten et al., 2000; Müller et al., 2002; Wei et al., 2002; Dyson et al., 2005; Muller-Gass et al., 2005; Sussman et al., 2005; Takegata et al., 2005). Because the MMN continues to be observed in this context, the MMN is considered relatively robust to manipulations of visual attention.

However, several studies suggest that the MMN may be reduced to task-irrelevant tones when subjects perform a difficult visual task that requires continuous vigilance. As reviewed below, the visual demands in these studies were tested at different levels of the same task. Specifically, subjects monitored a radar screen to detect and identify aircrafts during either low or high target density (Kramer et al., 1995), landed an aircraft during either low or high turbulence levels (Singhal et al., 2002), used a joystick to center a moving cursor that changed in velocity (low demand) or acceleration (high demand) (Yucel et al., 2005a, b), or monitored a rapid visual stream of letters for two numerals with the stimuli at either low demands (high contrast, long duration) or high demands (low contrast, short duration) (Haroush et al., 2010). The task-irrelevant tones were presented in an oddball task with frequency deviants. Critically, the tones were presented simultaneously with the visual stimuli to maximize competition between the task-relevant visual stimuli and the task-irrelevant tones. Thus, these studies used a stronger attention manipulation than tasks that allowed subjects to switch their attention between the visual stimuli and the tones (Haroush et al., 2010). A meta-analysis of these studies (with a total sample size of *N* = 69) suggested that as the difficulty of the visual task increased, the MMN decreased (i.e., the amplitude became less negative) (Wiens et al., 2016).

However, in two recent studies with duration deviants, we did not find a statistically significant effect of visual task difficulty on the MMN to the duration deviants (Wiens et al., 2016; Szychowska et al., 2017). In our studies, subjects searched for a target letter in a ring of letters that consisted either of one letter and five fillers (low load) or of six different letters (high load). This visual task is commonly used to manipulate perceptual load (Lavie, 2005). The irrelevant tones were presented at either 75 dB SPL (Wiens et al., 2016) or 65 and 55 dB SPL (Szychowska et al., 2017). When these two studies were combined (*N* = 83), the MMN was not significantly larger (i.e., more negative) for low load than high load, mean MMN difference = −0.31 μV, 95% CI [−0.65, 0.02] (Szychowska et al., 2017).

Nonetheless, when these two studies were combined with the previous studies on frequency deviants (Szychowska et al., 2017), the updated meta-analysis (*N* = 152) suggested that visual task difficulty decreased the MMN; the mean MMN difference (of low minus high) was −0.43 μV, 95% CI [−0.64, −0.22] with little evidence for heterogeneity of effect sizes (*I*^2^ = 14.52%). The homogeneity of effect sizes suggests that the type of oddball task (duration or frequency deviant) did not moderate the finding of no statistically significant effect for our recent studies versus a significant effect of visual task difficulty for previous studies.

At face value, the meta-analysis suggests that visual task difficulty decreases the MMN. However, the results of this meta-analysis need to be viewed with caution for several reasons (Wiens et al., 2016). Because previous studies had rather small sample sizes (between 9 and 28), these studies had low power and thus, a low sensitivity to obtain statistically significant results for a true effect (Stanley et al., 2018). Although alpha controls the rate of false positive, the low sensitivity to detect a true effect has the undesired consequence that a statistically significant result is more likely to be a false positive than a true positive (Ioannidis, 2005; Yarkoni, 2009; Ingre, 2013).

Furthermore, even if a statistically significant result reflects a true positive, the true effect size is necessarily overestimated in the context of low power (i.e., winner’s curse) (Ioannidis, 2008; Button et al., 2013). Specifically, if a study is unlikely to yield statistical significance for the true effect size (i.e., low power), the obtained effect must have been much larger than the true effect size to yield significance anyway. Accordingly, the meta-analytic estimate of low-powered (small-sample) studies overestimates the true effect.

Another issue is that a funnel plot of previous studies in other labs was largely biased toward one direction (favoring effects of visual task difficulty) and lacked studies that showed either no effect or an effect in the opposite direction (Wiens et al., 2016). Although formal statistical tests of this bias require a large number of studies (Sterne et al., 2011), the funnel plot suggests that effect sizes of previous studies were not distributed symmetrically around the mean effect size, as would be expected if only chance contributed to differences among the observed effect sizes. Thus, non-significant findings appear lacking for previous studies, and this absence of non-significant findings may be explained by a general bias by journals to publish significant rather than non-significant results (i.e., publication bias) (Bakker et al., 2012). So, even if the published studies are conducted well, a meta-analysis of only these studies will be biased because unpublished studies are not included. Furthermore, research suggests that in the presence of publication bias and other biases, there is no accepted procedure that corrects meta-analyses for these biases (Ioannidis, 2010; Inzlicht et al., 2015; van Elk et al., 2015; McShane et al., 2016).

Because previous results may be biased, the first goal of the present study was to provide new, unbiased data on whether visual task difficulty decreases the MMN. To strengthen the evidential value of our data, we preregistered hypotheses, method, and analyses (Nosek et al., 2018). This avoids any data-driven hypothesizing and minimizes biases from *post hoc* selection of electrodes and intervals (Kerr, 1998; Simmons et al., 2011; John et al., 2012; Luck and Gaspelin, 2017). Furthermore, Bayesian hypothesis testing was conducted instead of traditional null hypothesis testing (Dienes, 2014; Wiens and Nilsson, 2017; Wagenmakers et al., 2018b). Because the Bayes factor (BF) captures the relative evidence for the null hypothesis versus an alternative hypothesis, it can provide evidence for no effect. Also, Bayesian confidence intervals allow conclusions about the plausible size of the true effect.

The second goal of the present study was to reduce the confounding effect of physical differences between the tones on the MMN. In the oddball task, standard and deviant tones are by definition physically different and not presented equally often. Therefore, the neural responses will vary regardless of pattern regularity. These differences strongly affect the auditory N1: a frontocentral negativity with a latency of around 100 ms in response to physical changes in sound, including onset, offset, pitch, and intensity (Näätänen and Picton, 1987; Pratt, 2011). Because the N1 has a similar latency and topography as the MMN, an apparent frontocentral negativity may actually indicate effects only of these differences rather than of an unexpected auditory change (May and Tiitinen, 2010).

To reduce these confounding effects, Schröger and Wolff (1996) suggested a separate control condition that is sometimes referred to as the *equiprobable* condition: The critical tone is physically identical to the deviant, is presented as many times as the deviant, and is equiprobable with other tones (Schröger and Wolff, 1996). For example, the critical tone may be presented in the control condition together with nine other tones at a probability of 10% each. Trial order is pseudorandomized with the restriction that tone repetitions are avoided. Compared to a deviant that is presented on 10% of the trials in the oddball condition, the critical tone would be physically identical and presented as often. As a result, any obvious confounds from physical differences are eliminated between deviant and critical tone. A *corrected* MMN can be obtained by taking the response to the deviant in the oddball condition minus the response to the critical tone in the control condition (i.e., deviant minus critical). This corrected MMN has been used to study pattern violations in frequency (Jacobsen and Schröger, 2001; Jacobsen et al., 2003b), location (Schröger and Wolff, 1996), duration (Jacobsen and Schröger, 2003), and intensity (Jacobsen et al., 2003a).

Therefore, we recorded the corrected MMN as well as the oddball MMN to obtain a more direct measure of effects of visual task difficulty on pattern violation. Furthermore, because of concerns that previous findings of a reduced oddball MMN may have been caused by effects on the N1, we also analyzed whether visual task difficulty generally reduced the N1. To that end, mean N1 amplitudes across all tones in the control condition were compared between the two levels of visual task difficulty.

The third goal of the present study was to manipulate visual task difficulty while avoiding confounding effects from physical differences of the visual stimuli. In all previous studies, the visual stimuli differed physically between the levels of the task. For example, subjects monitored a rapid visual stream of letters that were shown either for a long duration at high contrast (low demand) or for a short duration at low contrast (high demand) (Haroush et al., 2010). Similarly, in our previous studies with letter rings, one condition used one letter and five fillers and the other condition used six letters (Wiens et al., 2016; Szychowska et al., 2017). Because physical stimulus differences generally have strong effects on ERPs (Luck, 2014), these physical differences may have confounded the MMN. A recent study was partly designed to address this question (Wiens et al., 2018). We recorded the oddball MMN and the corrected MMN while subjects performed a simple detection task on a circle at fixation. Simultaneously, task-irrelevant letter rings were shown either with one letter and five fillers or with six letters, as in our previous studies (Wiens et al., 2016; Szychowska et al., 2017). Results (*N* = 40) did not provide evidence for or against an effect of the number of letters on the MMN, that is, the confidence intervals overlapped zero and were wide (Wiens et al., 2018). Nonetheless, both oddball MMN and corrected MMN were numerically larger during the six-letter condition than during the one-letter condition. For the oddball MMN, the one-letter MMN was −2.29 μV and the six-letter MMN was −2.63 μV. For the corrected MMN, the one-letter MMN was −0.92 μV and the six-letter MMN was −1.58 μV. Thus, the mean difference of six-letter condition minus one-letter condition was −0.34 μV for the oddball MMN and −0.66 μV for the corrected MMN. If this represents the true effect, its direction would be opposite to that hypothesized for that of visual task difficulty when the letter ring is task relevant. Specifically, when the letter ring is task relevant, the difference between high load (i.e., six-letter condition) and low load (i.e., one-letter condition) should be positive rather than negative. Accordingly, it is possible that in our previous studies in which the letter ring was task relevant (Wiens et al., 2016; Szychowska et al., 2017), an effect of letter ring *per se* may have canceled out an effect of visual task difficulty. In the present study, we used identical visual stimuli in both levels of the visual task and manipulated only task instructions. In both low and high levels, the visual stimuli were crosses in different colors. During low load, targets were red crosses (upright or inverted), and during high load, targets were upright yellow and inverted green crosses. This conjunction task is a prototypical task to manipulate load and has been widely used in behavioral and neuroimaging studies (Lavie, 2005; Schwartz et al., 2005; Parks et al., 2011). Critically, because the visual stimuli were identical during low and high load, this task avoids any confounding of the ERPs from physical differences in the visual stimuli.

The fourth goal of our study was to examine the role of working memory capacity (WMC) in moderating effects of visual task difficulty on the N1, the oddball MMN, and the corrected MMN. Compared to individuals with low WMC, individuals with high WMC should be better able to keep attention on visual task-relevant information and should be less likely to be distracted by irrelevant tones (Unsworth and Engle, 2007). In support of this idea, high WMC apparently reduces behavioral distraction to deviant sounds (Unsworth and Engle, 2007; Hughes, 2014). For example, participants performed visual n-back tasks at low and high load in the context of irrelevant background speech (Halin et al., 2015). On a surprise memory test about the content of the background speech, individuals with low WMC showed a performance decrease from low to high load whereas individuals with high WMC had similar performance during low and high load. Thus, high WMC was associated with a smaller decrease in distracter processing from low to high load. In line with these results, we predicted that individuals with low WMC would exhibit a larger effect of load on N1 and MMN than would participants with high WMC. Whereas individuals with low WMC would be less distracted during high than low load, individuals with high WMC would not be distracted during low and high load because of better attentional control.

The fifth goal of the study was to explore the effect of load on auditory P3a. This response is typically evoked by deviants in the oddball task, is characterized by a central-parietal positivity about 300 ms after tone onset, and is believed to reflect involuntary attention switching to the stimuli (Escera and Corral, 2007; Polich, 2007; Horváth et al., 2008). Cognitive load has been shown to reduce P3a, but this has been mainly studied with intramodal load tasks (Berti and Schröger, 2003) or with auditory stimuli preceding the visual stimuli (Escera et al., 2003; SanMiguel et al., 2008). The current design presented an opportunity to explore whether P3a to auditory deviants would also be reduced in a task with concurrent presentation of auditory and visual stimuli. We reasoned that if load reduces N1 and MMN, then it should also reduce P3a.

Finally, after collecting the data, we realized the value of an exploratory (not preregistered) analysis of the effects of load on the P3 to visual targets. Because a software limitation did not allow us to record behavioral data (as explained below), we used the visual P3 to provide direct support for a visual load effect in the present study. The visual P3 was measured in response to the task-relevant visual stimuli whereas the auditory P3a was measured in response to the task-irrelevant tones. The visual P3 was used to index increased attention to the visual targets compared to non-targets (Polich, 2007). Visual P3 to targets (vs. non-targets) should be smaller during high than low load, possibly indicating reduced certainty or reallocation of cognitive resources (Kok, 2001; Watter et al., 2001; Gomarus et al., 2006; Hagen et al., 2006). Consequently, reduced visual P3 to targets would indicate that the task is more cognitive demanding.

In sum, the present study was primarily designed to provide unbiased evidence on whether visual task difficulty decreases the auditory MMN to frequency deviants. Specifically, the main goal was to examine if continuous visual task demands that vary between high and low load within the same task can affect the frequency MMN (as measured by EEG) to task-irrelevant tones that are presented simultaneously with the visual stimuli. The corrected MMN as well as the oddball MMN were measured to reduce confounding effects of physical differences between the tones. The visual task was a standard manipulation of perceptual load and avoided confounding effects of physical differences between the visual stimuli. WMC was also recorded to study if attentional control is a moderator. To ensure methodological rigor, hypotheses, method, and analyses were pre-registered.

## Materials and Methods

The study was preregistered before any data were collected (osf.io/ewg9x). Deviations from the preregistration are noted below. All data and scripts are available at a university depository (Wiens et al., 2019).

### Participants

Participants were recruited by using billboard notices on the Stockholm University campus and online billboards. Whereas the preregistration stated that recruitment would stop by the end of April 2018, the evidence was insufficient by that date. Therefore, we continued recruitment until the BF exceeded 3 or was below 1/3 for the primary hypotheses (see below). Participation was rewarded by completion of a mandatory course requirement or by a cinema ticket. The study was carried out in agreement with Swedish legislation. The Ethical Review Act regulates research involving humans. It does not require explicit ethical approvals unless the law applies to a study. In this study, participants performed a visual task with background sounds while electroencephalography was recorded. No sensitive personal information was collected. Thus, no ethics approval was required as per national regulations and university regulations. All subjects gave written informed consent in accordance with the Declaration of Helsinki.

All participants fulfilled the preregistered inclusion criteria (between the age of 18 to 40, no history of neurological diseases, normal or corrected-to-normal vision, and normal hearing). All participants had hearing levels within the normal range (i.e., less or equal to 20 dB), as confirmed by pure-tone audiometry at 500, 750, and 1000 Hz. Because of equipment malfunctioning, one participant was excluded because no EEG data were recorded. The final sample consisted of 49 participants. Of these participants, 22 were male, seven were left-handed and one ambidextrous, and their age ranged from 19 to 40 years (*M* = 27.7, SD = 5.67).

### Materials and Apparatus

Auditory stimuli were tones presented at 70 dB SL with in-ear tubephones (ER2; Etymotic Research Inc., IL^1^). Visual stimuli were crosses that were shown at a 3.2° × 3.2° viewing angle. A Cedrus StimTracker (Cedrus Corporation, San Pedro, CA, United States) was used to mark stimulus onset. The onset of the tones was indicated by the audio input to the headphones, and the onset of the crosses was indicated by a photodiode in the corner of the screen (where a white square appeared concurrently with the crosses).

### Procedure

Electroencephalography activity was recorded while participants completed a visual search task, which was modeled after the task in Parks et al. (2011). On each 500-ms trial, a cross was shown in the center of the screen for 100 ms. Crosses varied in orientation (upright or inverted) and color (red, blue, green, yellow, or violet). Participants were asked to press the space key on a keyboard as quickly as possible when they detected a target cross. In the *low* visual load condition, the targets were red crosses irrespective of orientation (upright or inverted). In the *high* visual load condition, the targets were upright yellow and inverted green crosses.

Both visual load conditions were presented in different blocks. Each block consisted of 360 trials, which comprised 72 targets and 288 non-targets (i.e., 20% were targets). The sequence of crosses was random with the restriction that the number of non-targets between targets ranged between two and six. Separately for targets and non-targets, the various combinations of color and orientation were presented equally often and in random order. An additional seven non-target trials before each block were drawn randomly from the eight possible non-target combinations. Thus, each block lasted about 3 min (367 × 0.5 s).

Simultaneously with the crosses, tones were presented for 100 ms. In the *oddball* condition, the tones were either 500 Hz (deviant) or 550 Hz (standard). Of the tones, 12.5% were deviants. The order of the tones was randomized with the restriction that there were at least three standards between two deviants. Before the occurrence of the first deviant per block, seven standards were presented (but excluded in the data analysis). In the *control* condition, the tones were 500, 550, 605, 666, 732, 805, 886, and 974 Hz. The order of the tones was randomized within each set so that the same tone was not presented twice in a row between sets (Wiens et al., 2018). Before the occurrence of the first 500-Hz tone per block, the other seven tones were presented (but excluded in the data analysis). For both tone conditions, 20% of the trials were visual targets for both the 500-Hz tone and the remaining tones. Thus, tone frequency did not predict visual targets.

Each block was one of the combinations of visual load (low or high) and tone (oddball or control). These four conditions were presented in random order twice for a total of eight blocks. Within each set of four conditions, condition order was randomized for each subject. Participants were instructed to ignore the tones while responding to the target crosses.

After the EEG recording, working memory capacity was measured with an operation span task (OSPAN) (Conway et al., 2005) by using py-span-task software (von der Malsburg, 2015). Participants were required to remember sequences of letters. After each 1-s letter, they were required to read out loud a mathematical expression, such as (5 + 7)/2 = 8, and judge its correctness with button presses. The expression was presented until participants responded or time ran out (the time limit was adjusted for each participant during a practice session). At the end of a letter sequence, participants were instructed to type in the letters in their correct order on a keyboard. Sequence length varied between 2 and 6 letters. Each sequence length was used three times. The order of the sequence lengths was randomized over trials. Performance was scored using a strict serial recall criterion. Accordingly, a response was counted as correct only if the letter was recalled in the correct serial position. The proportion of correct responses was computed for each sequence, and the partial credit unit (PCU) score was calculated as the mean proportion across all sequences.

### Electroencephalography

Electroencephalography was recorded with an Active Two Biosemi System (BioSemi, Amsterdam, Netherlands). Data were recorded from four electrodes at standard 10/20 positions (Fpz, Fz, Cz, and Pz) and from the closest approximation of the standard mastoid positions that were available in our 64-electrode cap (P9 and P10). An electrode on the cheek was used to detect eye movements. Two system-specific positions served as the internal reference (common mode sense, CMS, which was located between PO3 and POz) and as the ground (driven right leg, DRL, which was between POz and PO4). An electrode on the tip of the nose was recorded for later rereferencing of the data. The EEG was sampled at 1024 Hz and bandpass filtered between 0.1 Hz (software filtering) and 104 Hz (hardware filtering). Data processing was conducted in Matlab and the toolbox FieldTrip (Oostenveld et al., 2011).

Epochs were extracted from 100 ms before tone onset to 500 ms after tone onset. This epoch length was preregistered for P3a, but we used it also for N1 and MMN (instead of the preregistered 400 ms after tone onset) to facilitate plotting. Each epoch was baseline-corrected by subtracting the mean amplitude of the 100-ms interval before tone onset. The data were rereferenced to the tip of the nose. Fpz was also referenced to the nose to detect vertical and horizontal eye movements. For each participant, the distribution of epochs in terms of their amplitude ranges (i.e., max minus min within each epoch) was visually inspected, and outlying epochs were removed. Cutoffs were adjusted individually to retain as many trials as possible while reducing the potential effects of outliers. Because inspection was blind to the condition (load and tone) of individual trials, this inspection avoided bias (Keil et al., 2014). After artifact rejection, all subjects retained at least 70% of the epochs in the primary analyses (i.e., in each high and low load for deviants and standards, and to all tones in the control condition). Thus, no subject was excluded according to the preregistered criteria. In the primary analyses, the mean number of epochs across subjects was at least 86.7%.

For N1, MMN, and P3a, mean amplitudes were computed separately for low and high visual load. We preregistered the following electrodes and intervals for N1, MMN, and P3a: N1 was defined as the mean amplitude of Fz and Cz between 75 and 105 ms after tone onset for all tones in the control condition. Mean MMN amplitudes were computed across Fz and Cz between 125 and 175 ms after tone onset. Because the MMN is a difference score, the oddball MMN was defined as the mean amplitude difference of the deviant (500 Hz) minus the standard (550 Hz) in the oddball condition, and the corrected MMN was the mean amplitude difference of the deviant in the oddball condition minus the critical tone (500 Hz) in the control condition. For the P3a, mean amplitudes were computed across Cz and Pz between 300 and 500 ms after tone onset to deviants minus standards in the oddball condition. We computed the difference scores of deviants minus standards (rather than taking mean amplitudes only for deviants, as preregistered) because a reviewer pointed out that difference scores should eliminate any potential confounds from visual ERPs (as further explained in the discussion).

### Statistical Analyses

Analyses were carried out by using Bayesian statistics (Dienes, 2014; Wiens and Nilsson, 2017; Wagenmakers et al., 2018b). The BF was used for model comparison. It represents a ratio of the likelihood of the data given one model (e.g., the alternative hypothesis) over the likelihood of the data given another model (e.g., the null hypothesis). BF_10_ expresses this likelihood ratio in terms of the alternative hypothesis over the null, and BF_01_ expresses this likelihood ratio in terms of the null over the alternative hypothesis. For example, BF_10_ = 3 means that the data are three times more likely given the alternative hypothesis than given the null hypothesis. Note that because BF_10_ = 3 is identical to BF_01_ = 1/3, we report the value (i.e., BF_10_ or BF_01_) that is easiest to comprehend.

The analyses tested preregistered hypotheses that are referred to as H1 to H5 in the preregistration. The primary, preregistered hypotheses were that N1 and MMN would be smaller (i.e., less negative) during high visual load than low visual load. According to the hypotheses (H1 in regards to N1 and H2 in regards to MMN), the load effect (high minus low load) should be positive because a small negative value during high load minus a large negative value during low load equals a positive value. Unfortunately, the preregistration was unclear on two points. First, the primary hypothesis for N1 (i.e., H1) referred incorrectly to “N1 to pitch change” rather than to N1 to the tones *per se*. However, the description in the remainder of the preregistration and the computation are correct by referring to the N1 across tones. Second, the primary hypothesis for MMN (i.e., H2) referred to MMN in general, but the subsequent description of the analysis mentions only the corrected MMN. However, we analyzed both because H2 clearly applies to both the corrected MMN and the oddball MMN.

Although our hypotheses were directional, all inferential tests were two-tailed. The alternative hypothesis was modeled as a uniform distribution with the limits defined as -1.5 and 1.5 μV, and the likelihood defined as a *t* distribution. The BF was computed with Aladins Bayes factor in R custom scripts (Wiens, 2017). Although the BF is a numeric value that provides a continuous measure of evidence, we used an interpretation scheme to facilitate verbal communication (Wagenmakers et al., 2018a). For example, 1 < BF < 3 is anecdotal evidence, and 3 < BF < 10 is moderate evidence. We also computed the 95% credible interval (with an uninformed prior) of the mean differences.

To address secondary hypotheses in regards to N1 and MMN, correlations were calculated between WMC and the difference scores (high minus low load) for N1 and MMN. Participants with low WMC were expected to exhibit a larger (i.e., more positive difference score) effect of load on N1 and MMN than would participants with high WMC. Thus, the correlation between WMC and the difference scores for N1 and MMN was predicted to be negative (preregistered as H3). We also tested the secondary hypothesis that P3a would be smaller during high than low load (preregistered as H4). That is, the difference score of high minus low load would be negative because a small positive value during high load minus a large positive value during low load would yield a negative value. Further, because participants with low WMC were expected to exhibit larger effects (i.e., more negative difference scores), the correlation between WMC and the P3a differences scores was predicted to be positive (preregistered as H5). For correlations, we preregistered to report the 95% credible intervals (with an uninformed prior). Instead, we computed the BF as well as the 95% credible intervals with the alternative hypothesis modeled as a flat prior (β = 1).

Because a software limitation prevented accurate recording of behavioral data, additional secondary hypotheses in regards to reaction times (preregistered as H6 and H7) could not be tested. However, in a subsequent study with improved software, we used the same visual task but presented a continuous amplitude-modulated tone at 500 Hz (Szychowska and Wiens, under review). Behavioral results showed clear effects of load: Subjects (*N* = 43) were faster and more accurate during low visual load than high visual load. The mean difference in reaction times was 131 ms (95% CI [121, 141]), and the mean difference in *d’* was 1.68 (95% CI [1.50, 1.85]). These results provide indirect support for an effect of visual load on performance.

To provide direct support for a visual load effect in the present study, we conducted an exploratory analysis of effects of load on the P3 to visual targets (Kok, 2001; Watter et al., 2001; Gomarus et al., 2006; Hagen et al., 2006; Polich, 2007). Because 20% of the trials were visual targets, we computed ERPs for targets and non-targets, separately for the two tone conditions (oddball and control) and for low and high load. To capture the visual P3, mean amplitudes were extracted between 300 and 500 ms after the onset of the crosses across electrodes Cz and Pz (thus, P3 was defined identical to the P3a to tones).

## Results

**FIGURE 1:**
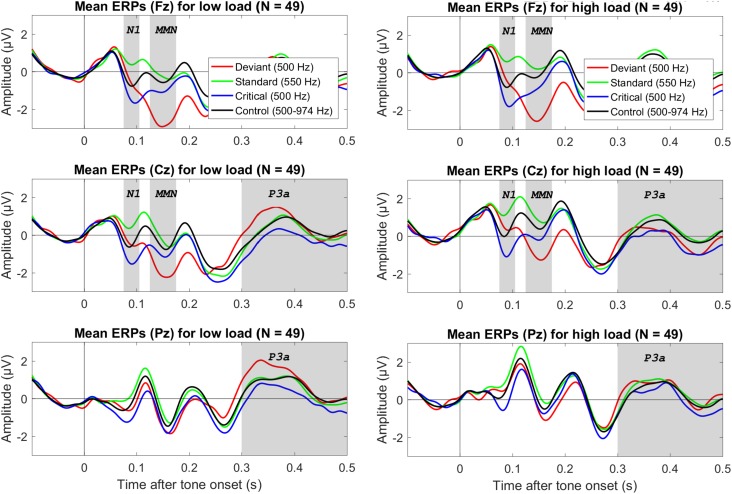
Grand average (*N* = 49) ERPs to the onset of different tones for three electrodes (in different rows), separately for low visual load (left column) and high visual load (right colum). The deviant and standard were from the oddball condition, the critical tone was identical to the deviant but from the control condition, and the control tone refers to all tones in the control condition. The gray bars mark the relevant electrodes and intervals for the N1, MMN, and P3a. The data were low-pass filtered at 30 Hz.

**FIGURE 2:**
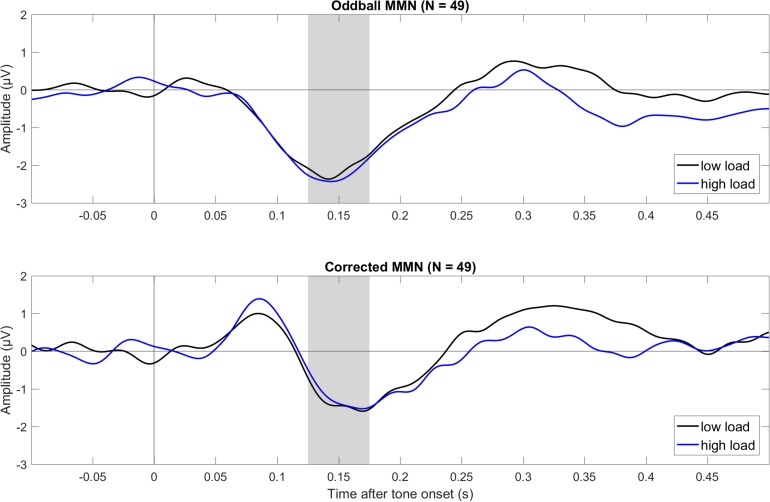
Grand average (*N* = 49) ERPs to tone onset for the oddball MMN (top) and corrected MMN (bottom) during low and high visual load (across Fz and Cz). The oddball MMN was the difference between deviant and standard in the oddball condition (deviant minus standard), and the corrected MMN was the difference between deviant in the oddball and the critical tone in the control condition. The gray bars mark the relevant intervals for the MMN. The data were low-pass filtered at 30 Hz.

Figure 1 shows mean ERPs for the different tones (deviant, standard, critical, and control) for three electrodes (Fz, Cz, and Pz) for low visual load (left) and high visual load (right). Figure 2 shows mean difference ERPs between deviant and standard tone (i.e., oddball MMN) and between deviant and critical tone (i.e., corrected MMN) for low and high visual load. Table 1 shows the descriptive and inferential statistics for the comparisons of interest. Note that the primary, preregistered analyses targeted effects of load on N1 and MMN in terms of the 95% CI and the BF with a two-tailed prior [−1.5,+1.5]. The remaining analyses were exploratory. For example, the BF[0,+1.5] captures the expectation that the effect of load is directional; that is, compared to low load, high load decreases mean amplitudes. All data, analysis scripts, and additional tables and figures (e.g., plots of the BFs and scatterplots with WMC) are available at the university depository (Wiens et al., 2019).

**TABLE 1:** Descriptive and inferential statistics for mean ERP amplitudes.

**ERP**	**Mean**	**95% CI**		**Mean**	**95% CI**		**Mean**	**95% CI**		**BF(−1.5,+1.5)**		**BF(0,+1.5)**	
								
	**Low load**	**LL**	**UL**	**High load**	**LL**	**UL**	**High-Low**	**LL**	**UL**	**BF01**	**BF10**	**BF01**	**BF10**
N1	–0.49	–1.02	0.03	–0.15	–0.72	0.41	0.34	0.04	0.64	0.67	1.50	0.34	2.95
Oddball MMN	–2.13	–2.73	–1.53	–2.26	–2.82	–1.71	–0.13	–0.74	0.47	3.57	0.28	5.56	0.18
Corrected MMN	–1.38	–2.15	–0.61	–1.26	–2.04	–0.47	0.12	–0.75	0.99	2.63	0.38	2.17	0.46
P3a (300 to 500 ms)	0.41	–0.34	1.16	–0.18	–0.89	0.53	–0.58	–1.59	0.42	1.25	0.80	0.71	1.40
P3a (300 to 400 ms)	0.68	–0.10	1.45	–0.08	–0.82	0.65	–0.76	–1.75	0.23	0.79	1.26	0.43	2.34
Visual P3	9.29	7.57	11.01	2.73	1.70	3.77	–6.56	–7.81	–5.30	0.01	201.39	0.01	400.97

*N1, MMN, and P3a refer to ERPs extracted in response to the tones (*N* = 49). Oddball MMN (and the P3a) is the difference between the deviant and standard in the oddball condition (deviant minus standard), and corrected MMN is the difference between the deviant in the oddball condition (i.e., 500-Hz tone) and the critical tone (i.e., 500-Hz tone) in the control condition (deviant minus critical). The visual P3 refers to the difference between visual targets and non-targets across oddball and control conditions. The primary, preregistered analyses targeted effects of load on N1 and MMN in terms of the BF with a two-tailed prior [−1.5,+1.5]. Note that for the analyses of BF [0,+1.5] for P3a and visual P3, the mean difference of low minus high was tested because this difference should be positive.*

The N1 to the control tones tended to be smaller (i.e., less negative) during high than low visual load. The mean difference of high minus low load was 0.34 μV, 95% CI [0.04, 0.64]. However, there was only anecdotal evidence for an effect (2.95 > BF_10_ > 1.50). Notably, although N1 amplitudes during either low or high load did not correlate directly with WMC (0.11 > *r* > −0.08), the load effect (i.e., high minus low load) on N1 tended to correlate positively with WMC; *r* = 0.35, 95% CI [0.08, 0.58], BF_10_ = 3.53 (see the university depository for a scatterplot). Accordingly, there was moderate evidence that the N1 decrease from low to high load was larger for participants with high than low WMC.

As shown in Table 1 and Figure 2, both the oddball MMN and the corrected MMN were apparent for both low load and high load. Mean amplitudes of the MMN were between −1.26 and −2.26 μV, and the estimated minimum effect size was −0.47 μV (as suggested by the upper limit of the 95% CIs).

Critically, results suggested no effect of load on MMN. For oddball MMN, results provided moderate evidence for no effect of load (5.56 > BF_01_ > 3.57). For corrected MMN, results provided only anecdotal evidence for no effect of load (2.63 > BF_01_ > 2.17). Because MMN is often recorded with a mastoid reference, we conducted an exploratory analysis on the effects of load on the MMN when the data were rereferenced to the mean of P9 and P10, which we recorded as the closest neighbors of the mastoids, rather than to the tip of the nose (as preregistered). These supplementary analyses are available at the university depository. Results provided moderate evidence for no effect of load, both for oddball MMN (9.09 > BF01 > 5.56) and for corrected MMN (4.55 > BF01 > 4.00). Thus, results suggested no effect of load on MMN independent of the reference electrode.

Further, WMC did not correlate with effects of load on the oddball MMN; *r* = −0.21, 95% CI [−0.46, 0.08], BF_01_ = 2.02. Similarly, WMC did not correlate with effects of load on the corrected MMN; *r* = −0.12, 95% CI [−0.39, 0.16], BF_01_ = 3.94.

Analyses of the P3a to deviants (versus standards) did not suggest an effect of load for the preregistered interval between 300 and 500 ms (see Table 1). Although visual inspection of Figure 1 suggested that the P3a to deviants (versus standards) tended to be larger during low load than high load between 300 and 400 ms after tone onset, an exploratory analysis of the mean amplitudes during this interval provided only inconclusive evidence (see Table 1). WMC did not correlate with effects of load on P3a; −0.11 > *r* > −0.12, 4.20 > BF_01_ > 3.95.

**FIGURE 3:**
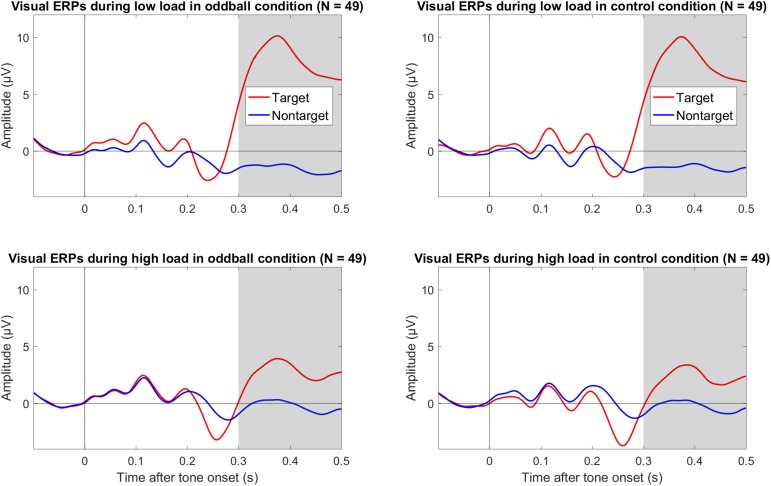
Grand average (*N* = 49) ERPs to onset of the visual targets and non-targets in the oddball condition (left column) and the control condition (right column) during low visual load (top row) and high visual load (bottom row) across Cz and Pz. The gray bars mark the relevant intervals for the visual P3. The data were low-pass filtered at 30 Hz.

We also explored effects of load on the P3 to visual targets. As shown in Figure 3, there was a large positivity to targets compared to non-targets. Importantly, this positivity to targets versus non-targets was smaller during high load than low load, and this pattern was similar in both tone conditions (oddball and control). As shown in Table 1, the mean amplitude difference between targets and non-targets across both tone conditions was more positive during low load than high load. The mean difference of low minus high load was 6.56 μV, 95% CI [5.30, 7.81], and the BF_10_ > 201 provided extreme evidence for an effect (see Table 1). There was no evidence that load effects on the visual P3 differed between tone conditions; the mean difference between oddball and control was 0.31 μV, 95% CI [−0.69, 1.31].

## Discussion

In this large sample (*N* = 49), an MMN was obtained in all conditions, that is, for the oddball MMN and the corrected MMN in both low and high visual load (see Figure 2 and Table 1). Critically, evidence suggested that visual load did not affect the MMN. With the preregistered reference electrode (i.e., tip of nose), the strength of evidence for no effect of load was moderate for the oddball MMN and anecdotal for the corrected MMN. With the mastoid reference (i.e., mean of P9 and P10), the strength of evidence for no effect of load was moderate for both the oddball and the corrected MMN. Furthermore, evidence suggested there was no correlation of working memory capacity with effects of load on the oddball MMN and the corrected MMN.

Several previous studies with small sample sizes reported a statistically significant decrease of the oddball MMN during a concurrent difficult visual task (for review, see Wiens et al., 2016). However, a funnel plot of these studies suggested a lack of studies with either no effect or an effect in the opposite direction, as would be expected by chance. Also, more recent studies obtained only statistically non-significant findings despite larger sample sizes (Wiens et al., 2016; Szychowska et al., 2017). Last, a recent meta-analysis of previous studies (Wiens et al., 2016) does not provide convincing evidence because current meta-analytic procedures are unable to fully correct for systematic biases (Ioannidis, 2010; Inzlicht et al., 2015; van Elk et al., 2015; McShane et al., 2016).

In this context, the preregistration of the present study minimized any potential biases from data-driven hypotheses and flexible analysis strategies such as *post hoc* selection of electrodes and intervals (Kerr, 1998; Simmons et al., 2011; John et al., 2012; Luck and Gaspelin, 2017; Nosek et al., 2018). Therefore, the present results are important because they provide an unbiased estimate of the effect of load. This unbiased estimate is that the oddball MMN is robust to manipulations of visual load.

In the oddball MMN, the response to a tone (deviant) is compared to the response to a different tone that is presented more often (standard). Thus, responses to these tones may differ simply because of physical differences and not because of an unexpected auditory change. Specifically, deviant and standard differ from each other and are not presented equally often. Because the N1, which has a similar latency and topography as the MMN, is strongly affected by these physical differences, the oddball MMN may be confounded by larger N1 responses to the deviant than standard (Näätänen and Picton, 1987; May and Tiitinen, 2010; Pratt, 2011). To reduce the confounding effects of these physical differences, the present study included a separate control condition (Schröger and Wolff, 1996). The critical tone was the same tone as the deviant. It was presented as many times as the deviant in the oddball condition and was presented equally often as seven other tones (i.e., 12.5% each). Thus, any obvious confounds from physical differences were eliminated when comparing the deviant tone in the oddball condition with the critical tone in the control condition. As shown in Figure 2 and Table 1, the present results suggested that this corrected MMN was not affected by load. Although the strength of evidence was only anecdotal with the preregistered reference electrode (i.e., tip of nose), it was moderate with a mastoid reference (i.e., mean of P9 and P10).

Because in both load conditions, the visual stimulation was identical, any potentially confounding effects from visual differences between the load conditions were eliminated. This is a beneficial feature of this task because in a previous study in which the visual load conditions differed in the visual stimuli, results suggested that the oddball MMN and the corrected MMN may be confounded by these visual differences (Wiens et al., 2018). Therefore, the present study is the first to assess effects of visual load while avoiding confounding effects from physical differences of the visual stimuli.

Because the oddball MMN may be confounded by effects of physical differences on the N1, the present study also measured effects of visual load on the N1. As shown in Table 1, there was some evidence that high load decreased the N1. Unfortunately, the design of the present study does not rule out that these load effects on the auditory N1 might have been mediated by load effects on the visual N1. Because visual stimuli were presented concurrently with the tones, it is possible that high load reduced the visual N1 rather than the auditory N1. If this effect spread to the electrodes that we used to record the auditory N1, the auditory N1 across all tones in the control condition would be confounded. In contrast, the results of the MMN (as well as the P3a to tones) are unaffected: Because these ERPs were always computed from a difference wave between tones during a particular load, any confounding effects from the visual ERPs on the auditory ERPs would have affected the ERPs to both tones and would thus have been eliminated in the difference wave.

Although there was no apparent evidence for a load effect on P3a in the preregistered interval (300 to 500 ms after tone onset), Figure 1 suggested that P3a tended to be larger during low than high load between 300 and 400 ms after tone onset. However, an exploratory analysis of this interval provided only inconclusive evidence that high load decreases the P3a (see Table 1). Whereas previous studies in which the auditory stimuli preceded the visual stimuli found that cognitive load decreased the P3a to auditory deviants (Escera et al., 2003; SanMiguel et al., 2008), the present results do not resolve whether high visual load decreases attentional capture by auditory deviants in a context with simultaneous presentation of auditory and visual stimuli.

WMC did not correlate with load effects on the oddball MNN and the corrected MMN. The strength of evidence was anecdotal for the oddball MMN and moderate for the corrected MMN. Also, there was moderate evidence that WMC did not correlate with P3a. In contrast, there was moderate evidence that WMC correlated positively with load effects on the N1. Accordingly, the N1 decreased more strongly from low load to high load for individuals with high WMC than for individuals with low WMC. This result contradicts our prediction that load effects would be larger for individuals with low WMC. However, because load effects on the auditory N1 might potentially be confounded by load effects on the visual N1 (as explained before), this result has limited value. Accordingly, the present results raise doubts about the claim that WMC moderates effects of load. Instead, the present findings are consistent with recent evidence in a large sample (*N* = 601) for no relationship between WMC and either the changing state effect or the deviation effect in serial recall (Korner et al., 2017). In sum, results do not support the idea that individuals with high WMC are less distracted by task-irrelevant stimuli than are individuals with low WMC (Unsworth and Engle, 2007).

The present findings suggest that the frequency MMN is unaffected in a continuous visual task in which demands vary between high and low load and the tones to elicit the MMN are presented simultaneously with the visual stimuli. However, this conclusion should be qualified by some considerations. First, a possible explanation for the absence of a load effect is that this particular load manipulation is not strong enough. However, the present task has been advocated as a prototypical manipulation of perceptual load (Lavie, 2005; Schwartz et al., 2005; Parks et al., 2011), although this task may load cognitive as well as perceptual processes (Murphy et al., 2017). Although behavioral data could not be obtained in the present study (because of a software limitation), we used the same task in another study with a continuous amplitude-modulated tone at 500-Hz as a distracter and found strong effects on performance (Szychowska and Wiens, under review). In the present study, we also explored effects of visual targets (vs. non-targets) on the visual P3 (Hagen et al., 2006; Polich, 2007). Results provided extreme evidence that the visual P3 was reduced under high visual load (see Figure 3 and Table 1), supporting the idea that high load was more difficult than low load (Kok, 2001). Nonetheless, because there is no evidence for the claim that the present load manipulation is stronger (or weaker) than that used in other studies, the present findings cannot be generalized beyond that of the present load manipulation. Accordingly, the present findings imply only that load manipulations similar to that in the present study should not affect the MMN.

Second, it is unresolved if the conclusion is valid for other task designs and measures. For example, the present findings do not resolve whether the present load manipulation affects the frequency MMN in complex stimulus sequences (Nordby et al., 1988; Saarinen et al., 1992; Alain et al., 1994; Paavilainen et al., 1995; Paavilainen, 2013). Also, the present findings were obtained for the frequency MMN, and it is unresolved whether other types (such as duration and intensity MMN) are also insensitive to load effects. Last, whereas the present study used a crossmodal manipulation of attention, evidence suggests that intramodal manipulations of attention affect the MMN (Woldorff et al., 1991; Alain and Woods, 1997).

To conclude, the present findings support the robustness of the auditory frequency MMN to manipulations of visual attention and suggest that this relationship is not moderated by working memory capacity.

## Data Availability

All datasets generated for this study are included in the manuscript and/or the supplementary files.

## Author Contributions

All authors designed the study together. RE and MS programmed the experiments and analyzed the behavioral data. MS and EB collected the data. SW analyzed the EEG data and conducted the Bayesian analyses. SW and EB wrote the manuscript.

## Conflict of Interest Statement

The authors declare that the research was conducted in the absence of any commercial or financial relationships that could be construed as a potential conflict of interest.

## Footnotes

1 www.etymotic.com
